# Navigating paradox in child welfare: implications for improvement science in complex human service systems

**DOI:** 10.3389/frhs.2026.1767083

**Published:** 2026-03-25

**Authors:** Christina Evaldsson, Sofia Kjellström

**Affiliations:** 1 The Jönköping Academy of Improvement and Leadership, Department of Quality Improvement and Leadership, Jönköping University, Jönköping, Sweden; 2 Social Services Department, Jönköping Municipality, Jönköping, Sweden

**Keywords:** child welfare, complex human service, improvement science, paradox, quality, quality improvement, social work

## Abstract

**Introduction:**

Improvement science emphasizes the need to understand and enhance quality in complex service systems. While the field primarily has developed within healthcare, its expansion into social care requires deeper engagement with how quality is understood and enacted in practice. This study addresses this need by exploring how child welfare professionals in Sweden understand and enact “quality” in their everyday work with implications for improvement science in complex human service systems.

**Methods:**

The study employed a qualitative, practice-oriented, and interactive research design. The data consisted of 28 semi-structured interviews with professionals in child welfare, an analysis seminar, and documents (such as a quality management system and quality reports). An inductive content analysis was conducted, constructing themes through systematic interpretation rather than predefined frameworks.

**Results:**

The analysis identified two coexisting logics of quality: one emphasizing uniformity, the other emphasizing responsiveness to uniqueness. The findings show how professionals move between these logics as tensions become salient in daily practice.

**Discussion:**

By conceptualizing quality as a paradoxical construct, the study highlights how quality in child welfare is enacted through the ongoing negotiation of multiple logics, with implication for improvement science. The findings align with previous research suggesting that sustainable improvement involves the interplay of generalizable and contextual knowledge, and that paradoxes in organizational life shape conditions for learning and improvement.

**Conclusion:**

Quality in child welfare is not a fixed attribute but a negotiated phenomenon, shaped by persistent tensions between uniformity and responsiveness. Recognizing these paradoxes invites reflection on how improvement science engages with quality in complex human service systems, particularly in relation to the interplay between codified standards and professional judgment.

## 1 Introduction

Social welfare systems face mounting challenges driven by complex social and economic problems, making the delivery of high-quality care for children and families more critical than ever. Improving outcomes for those who rely on these services requires systematic approaches to quality, yet improvement frameworks have been applied to only a limited extent in social care. At the same time, improving quality in welfare services entails navigating persistent and competing demands that cannot be resolved through technical solutions alone. Improvement science, which was originally rooted in industrial contexts and later adapted to healthcare, offers structured methods for learning and change, but its application in social welfare remains underdeveloped. Paradox theory offers a complementary perspective by conceptualizing such demands as enduring features of organizational life rather than problems to be eliminated. Viewing welfare as a complex system foregrounds how such tensions and contradictions are embedded in everyday professional practice. Change, diversity, and resource constraints intensify experiences of tensions and paradoxes (1). This article explores these dynamics by examining how professionals perceive quality in child welfare, drawing on paradox theory, with implications for how improvement science engages with quality in complex human service systems.

### 1.1 Child welfare as a complex setting and an arena for quality

Social work, and child welfare in particular, constitutes a compelling setting for advancing theoretical insight. In the Swedish context, child welfare services provide a wide range of interventions, from supportive measures such as family counselling to compulsory actions including foster care and residential care, aimed at addressing both maltreatment and behavioral problems (2). These services operate within a legal framework that requires practitioners to balance support and control (3) at the same time as policy trends towards standardization create tensions between relational engagement and procedural consistency (4, 5). These services are characterized by high levels of uncertainty and ethical complexity, often involving emotionally demanding situations and high-stakes decision making. This complexity shapes the very meaning of quality in child welfare.

Research on quality in social work underscores its relational, processual, and situated character. High-quality practice depends on social workers' interpretive capacity, ethical judgment, and ability to build responsive, trust-based relationships with service users (6–8). From this perspective, quality is not merely an outcome but an unfolding practice shaped by the interactions between professionals, families, and organizational conditions. Taken together, these insights highlight that quality in social work cannot be fully codified in guidelines or checklists but instead emerges through situated reasoning and relational engagement. At the same time, governance models such as New Public Management have strongly influenced the development of quality in public services, including child welfare, by emphasizing efficiency, standardization, and measurable outcomes (4, 9). This has created tensions between bureaucratic logics on the one hand and professional logics that value autonomy and contextual responsiveness on the other. Rather than being resolved, these tensions persist and shape everyday practice, making quality work in child welfare a balancing act between competing demands. In Sweden, systematic quality improvement is mandated by the Social Services Act (SFS 2025:400) and further regulated through SOSFS 2011:9, a national set of binding regulations and guidelines outlining requirements for management systems for systematic quality work. Yet, despite this regulatory framework, approaches to improvement have largely been framed by governance ideals and organizational routines rather than by a coherent theoretical foundation. This disconnect underscores how child welfare is not only a complex arena for defining and enacting quality but also a critical context for examining how improvement efforts can be adapted to the realities of human service work, making this a particularly rich setting to examine how quality is understood and enacted within complex human service systems.

### 1.2 Improvement science

Improvement science has emerged as an interdisciplinary field that combines methodological and scientific rigor with a focus on understanding how quality can be created, supported, and sustained in complex service systems. One of the key questions for improvement science today is: “How can we improve the way we improve?” (10, p. 3). Improvement practices are typically applied in contexts characterized by complexity, such as health and welfare systems, where multiple, often conflicting goals coexist. While many improvement methods have their roots in industry and in Deming's work on systems thinking and statistical process control, improvement science as a distinct field of inquiry has primarily been developed within healthcare, drawing on organizational theory, psychology, systems thinking, and statistical methods to support systematic and practice-based change (11–14). Improvement science bridges research and practice by combining scientific theories with practical know-how (15). Despite growing interest in applying improvement science beyond healthcare—to social care, education, and community services—its theoretical foundations remain largely health-centric (16, 17).

This rootedness in healthcare raises important questions about how improvement science can be developed further to address the distinctive challenges of other complex human service contexts, drawing on insights from sectors beyond healthcare. Social work, for example, has been conceptually and empirically peripheral to this development. Few studies explicitly apply improvement science as a theoretical lens in social work [see, e.g., (18)], leaving a gap in understanding how improvement approaches intersect with the relational and situated nature of quality in child welfare. Traditionally, improvement science has approached tensions through a logic of resolution—seeking alignment and reducing variation, often assuming that contradictions can be solved through standardization and procedural consistency. While these strategies have advanced reliability, they risk oversimplifying the complexity of human service work, where competing demands persist. Scholars have increasingly highlighted the need to balance generalizable knowledge with sensitivity to the contextual, relational, and situated nature of service delivery (12). Extending this line of thought, Batalden and Foster (19) propose a shift towards “Quality 3.0,” which represents a conceptual reorientation of quality from compliance with predefine standards towards a view of quality as emergent, relational, and co-produced in practice. In this perspective, quality is not primarily achieved through standardization alone, but through partnerships among professionals, service users, and other stakeholders, emphasizing collaborative learning and responsiveness to context. Building on Marshall et al.'s (20) characterization of improvement science as a pre-paradigmatic field, Reed et al. (10) argue that this field is still defining its scope, boundaries, and core concerns, within which perspectives such as “Quality 3.0” contribute to ongoing debates about how quality should be conceptualized and studied. In their overview of the improvement landscape, Reed et al. (10) propose four overarching features that collectively distinguish improvement science: its grounding in complex social systems, a holistic and practically oriented use of multiple forms of knowledge, a practical orientation towards real-world usefulness, and the co-production of change by multiple actors. These features can be seen as strengthening the field's relevance to real-world practice while also creating tensions, such as those between standardization and local adaptation and between generalizable evidence and contextual, experiential insight. Addressing such tensions is central to consolidating the field's conceptual foundations and enhancing its capacity to support improvement across diverse settings.

### 1.3 Paradox theory as a lens

Paradox theory provides a lens for examining how organizations manage persistent tensions. A paradox is defined as “contradictory yet interrelated elements that exist simultaneously and persist over time” (21, p. 382). These tensions involve persistent opposition between elements that are mutually dependent (22). Unlike dilemmas or trade-offs, paradoxes cannot be resolved by choosing one side over the other; they endure and require ongoing navigation (23, 24). Paradoxes arise when organizational actors confront demands that are logical on their own but contradictory when combined. These tensions may remain latent, being underlying and not immediately visible, or salient, becoming explicit challenges in practice (21, 23). Hahn and Knight (25) argue that paradoxes are both inherent and socially constructed. They exist as latent potential within organizational systems and become salient when actors engage with them in specific contexts. This means that paradoxes change from abstract tensions to concrete challenges through interpretation and action. According to Smith and Lewis (21), organizations face different kinds of paradoxes, such as those related to learning (exploration vs. exploitation), belonging (individual vs. collective identity), organizing (flexibility vs. control), and performing (competing goals).

Applying paradox theory to quality improvement is valuable because organizational change amplifies paradoxical tensions: “Since change, plurality, and scarcity intensify experiences of paradox, the time is ripe for paradox to inform our thinking, research, and leadership across a broad range of phenomena” (1, p. 26). This means that paradox theory becomes especially relevant in contexts of rapid change and complexity, such as quality improvement and social care, because these conditions make tensions more visible and harder to ignore.

Paradox theory offers a powerful lens for understanding tensions and interdependencies in complex systems. While the theory is widely used in organizational research to address dynamic interconnections, it remains largely unexplored within improvement science. Early works explored the challenge of balancing exploration and exploitation in organizational learning (26, 27), followed by studies on alignment and flexibility in strategic management (28) and stability and change in quality improvement contexts (29). More recent studies have extended paradox perspectives to quality improvement and welfare-related settings, highlighting tensions such as stability and change, efficiency and sustainability or inclusion, and standardization and responsiveness (30–33). These examples illustrate that paradoxes are persistent contradictions between interdependent elements that organizations must navigate over time. While paradox theory helps to illuminate persistent organizational tensions, it does not offer prescriptive solutions. In this study, the theory is used analytically to interpret how quality-related tensions are experienced and navigated in practice.

### 1.4 Research contribution and aim

While improvement science primarily focuses on systems, variation, and learning, social work research offers nuanced accounts of how quality is constructed and negotiated in relational and emotionally complex human service work. Despite this complementarity, these knowledge domains seldom intersect: improvement science has tended to pay limited attention to how quality is constructed in everyday practice, while social work research has rarely connected its insights to improvement science as a field. Bridging this divide is important for ensuring that approaches to improvement are grounded in the realities of diverse human service systems. Against this background, this article aims to explore how child welfare professionals in Sweden understand and enact “quality” in their everyday practice. Drawing on paradox theory, the study uses a paradox perspective to interpret how competing understandings of quality coexist in child welfare practice.

The primary contribution of this article lies in its empirical and conceptual insights to quality in child welfare practice. By identifying two coexisting logics of quality: one emphasizing uniformity, the other emphasizing responsiveness to uniqueness. These logics are not merely oppositional but constitute a persistent paradox that professionals must navigate in daily practice. By applying paradox theory (21, 22), this article reconceptualizes these tensions, not as problems to solve but as enduring contradictions that shape quality work. The findings raise implications for improvement science, particularly in relation to how quality is conceptualized in complex human service systems, and contribute to ongoing discussions about the theoretical scope of the field (10).

## 2 Method

### 2.1 Design

A qualitative, practice-oriented design with an interactive approach was adopted. Grounded in an interpretive qualitative tradition (34), this study explored professionals' understandings of quality, and how this is enacted in everyday child welfare practice. Particular attention was given to their assumptions, experiences, and actions. By foregrounding participants' lived experiences and voices, the study generated insights into the complexity and situated nature of quality. The design and reporting were guided by the COREQ principles (35).

An interactive research approach (36, 37), closely aligned with Van de Ven's (38) concept of engaged scholarship, shaped methodological decisions throughout the study. This approach assumes that complex organizational challenges cannot be adequately understood through detached observation alone; instead, they require systematic engagement with those who experience, enact, and attempt to resolve these challenges in practice. This resulted in early and continuous engagement with practitioners, beginning with exploratory conversations that informed the formulation of the research question by identifying a practice-based knowledge gap and ensuring relevance to everyday professional concerns. The interactive approach also influenced participant engagement during the analytical phase. Preliminary findings were discussed with practitioners in an analysis seminar, where participants reflected on the interpretations and contributed practice-based insights. These discussions were used to assess the credibility and resonance of the findings rather than modify categories or reach consensus. Through this process, knowledge was co-constructed in dialogue between research and practice, supporting both practical utility and scientific rigor. In the later stages of the study, particular emphasis was placed on collective learning from the findings and their potential implications for practice development. The research was conducted under the auspices of the Swedish National Research School for Practitioners in Social Services (FYS), which emphasizes the integration of research and practice. Data from a licentiate thesis (39) form the basis of the study.

### 2.2 Context and participants

The study was conducted within child welfare services in Jönköping municipality, Sweden. Participants were selected using purposive sampling (40) designed to capture diverse perspectives on quality across organizational levels and professional roles. Inclusion criteria were based on participants' involvement in quality-related activities and their position within the organization. Participants included both strategic professionals, such as managers and development leaders responsible for planning and quality assurance, and clinical professionals, such as social workers engaged in direct client work. Clinical professionals were further selected to represent both family support and foster care services. This distinction reflects the dual roles in child welfare: strategic actors contribute to shaping organizational directions, and clinical actors bring experiential insight that helps adapt these directions in practice. Demographic characteristics such as gender or age were not used as inclusion criteria, as the analytical focus was on professional roles, organizational context, and work practices rather than individual attributes.

Initial oral information about the study, including its purpose, design, and plans for feedback and dissemination of results, was provided to all professionals working in the child welfare services at a regular staff meeting. Following this general information, individuals were later invited via e-mail to participate. In total, 28 interviews were conducted: 11 with strategic professionals and 17 with clinical professionals. Among the clinical group, 8 worked in statutory roles involving assessments and decisions under the Social Services Act, while 9 were engaged in family treatment roles focusing on supportive interventions. Two additional professionals were invited but declined participation at short notice, citing perceived time constraints. The number of participants was determined by the aim of achieving sufficient variation and informational richness to address the research questions. Data collection continued until the material was judged to have sufficient informational power to address the research questions.

### 2.3 Data collection

Data was gathered through multiple sources. Semi-structured interviews were conducted with professionals to elicit their experiences and interpretations of quality. Consistent with the practice-oriented approach, interviews focused on concrete situations, and follow-up questions were adapted to participants' roles and organizational contexts. This flexibility allowed participants to guide the conversation toward aspects of quality they considered most salient in their daily work.

The interviews lasted on average 50 min (range: 28–75 min) and were conducted primarily in a digital format using Zoom due to pandemic-related restrictions; in all cases, only the interviewer and the participant were present. All interviews were conducted by the first author, who at the time of the study was a PhD student with prior professional experience in child welfare practice and quality improvement work.

Interviews and the analysis seminar were audio-recorded and transcribed verbatim. To enable triangulation and enhance credibility, additional data sources included organizational documents (such as quality management system, management statements, quality reports, activity reports, and strategic plans), which were collected and analyzed to provide contextual understanding and examine how quality was articulated in formal texts.

### 2.4 Analysis

A conventional content analysis (41) was performed following the approach described by Graneheim and Lundman (42), meaning that categories were derived directly from the data rather than imposed by pre-existing theory. This approach allowed for an open exploration of participants' perspectives without limiting interpretation to predefined frameworks. The process began with transcription and familiarization with the material, followed by identifying meaning units and coding. Codes were then grouped into subcategories and organized into three analytical categories: *Tools for understanding*, *Orientation for action*, and *Value creation*. Interviews and documents were analyzed in parallel, comparing codes across sources to identify patterns and contradictions. This iterative process allowed insights from documents to inform the interpretation of interviews and vice versa, strengthening the depth of our analysis. Triangulation across data sources and iterative discussions with academic colleagues and study participants enhanced trustworthiness.

In a final step, a higher level of abstraction was created to explore latent content (42) across categories, which led to the development of an overarching theme: *Quality means navigating uniformity and responsiveness to uniqueness*. This phase involved an interpretive analysis, in which paradox theory (21) was used as a sensitizing framework to inform the interpretation of tensions between standardization and individualization without guiding the initial coding or category construction. Paradox theory thus contributed to framing these tensions as interdependent rather than mutually exclusive.

### 2.5 Ethical considerations

According to Swedish law, this type of study, which does not involve sensitive personal data or health-related information, does not require formal ethical review. Nevertheless, all applicable research ethics laws (SFS 2003:460) and recommendations (43) were followed. To respect participants' autonomy and integrity, they were provided with detailed written and verbal information about the study's purpose and procedures as well as their rights. Informed consent was obtained prior to participation, and confidentiality was ensured by using pseudonyms in all data and by securely storing transcripts and documents. Participation was voluntary, and individuals were reminded of their right to withdraw at any time without consequences.

## 3 Results

The analysis resulted in three interrelated analytical categories that together illuminate how child welfare professionals understand and enact quality in their everyday work. Each category captures a distinct but overlapping aspect of how quality is interpreted, practiced, and negotiated in this context. The categories are presented in a narrative format, supported by illustrative quotations from the interviews and complemented by insights from organizational documents.

Taken together, the three analytical categories reveal a dual and paradoxical understanding of quality. Although analytically distinct, they should be understood as mutually reinforcing elements within the broader organizational discourse on quality.

The findings point to two coexisting logics of quality in child welfare practice: quality as uniformity and quality as responsiveness to uniqueness. These logics are not mutually exclusive but reflect a dynamic tension that professionals need to navigate in their daily work.

To structure the empirical material, three analytical categories were applied: *tools for understanding*, *orientation for action*, and *value creation*. These categories clarify how the two logics manifest in professional reasoning and practice.

Table 1 provides an overview of these categories and their associated characteristics. Quality understood as uniformity is grounded in generalizable knowledge, codified procedures, and the pursuit of political goals. When understood as responsiveness, quality is rooted in particularistic knowledge, adaptation, and the aim to create life-changing impact for the individual.

**Table 1:** Analytical structure of the dual logics of quality across three categories.

Analytical categories	Quality as uniformity	Quality as responsiveness to uniqueness
Tools for understanding	Generalizable knowledge	Particularistic knowledge
Orientation for action	Codified procedures	Adaptation
Value creation	Political goals	Life-changing impact for the individual

### 3.1 Tools for understanding

The first category, tools for understanding, refers to the knowledge resources professionals use to achieve quality in their work. These tools provide orientation when navigating complex situations. The professionals emphasized that quality cannot be reduced to a single form of knowledge but is shaped through a dynamic interplay between two types: generalizable and particularistic knowledge.

#### 3.1.1 Generalizable knowledge

The professionals described generalizable knowledge as a vital dimension of quality, forming a shared foundation for practice and supporting legitimacy. This knowledge includes understanding key domains such as violence, addiction, diagnoses, and applicable interventions, as well as the organizational structures that shape everyday work. One strategic professional illustrated this point:

And that is also a form of quality, what do we need to monitor based on developmental theories and what's good for children, and what is not good for children, and how we can compensate for parents who have certain shortcomings. (Strategic professional 6)

Such knowledge serves as a stable reference point, guiding actions grounded in scientific evidence and reinforcing confidence that practice aligns with recognized norms and best practices.

#### 3.1.2 Particularistic knowledge

At the same time, particularistic knowledge was seen as crucial for being able to address the unique needs of individuals. This knowledge is situated, relational, and rooted in attentiveness to context. The professionals described how planned conversations often change course in response to the client's immediate situation:

When you prepare for a conversation, you think, ‘I’m going to bring up this and this and that,' but when you enter the room, you don't bring it up because the person is somewhere else entirely, and then I'm flexible and follow their perspective. (Clinical professional 8)

Here, quality emerges as an interactive process rather than an objective standard. It involves creating an experience that feels empathetic and tailored to the individual's circumstances. One clinical professional noted:

No case is like another. […] you have to adapt to each person. The children I work with range from five to twenty-one [year-olds]. That alone requires completely different ways of approaching, interacting, and explaining. (Clinical professional 7)

#### 3.1.3 Navigating the duality

The findings indicate that these knowledge forms are not perceived as mutually exclusive but rather as complementary:

… there are basic conditions for what is good for children and so on, but it's not certain that this exactly matches what is best for this particular child. (Analysis seminar participant C)

This integration, however, is not without challenges. Research and evidence provide guidance but are rarely experienced as directly applicable:

We know from research studies that placements can introduce risks in children's lives. But also that children should be placed [in care] when there are serious problems because that is a precondition for being able to protect these children. […] So we're fumbling a bit. (Strategic professional 3)

Participants described how decisions grounded in research require interpretation and contextualization. Professionals referred to drawing on both forms of knowledge as tools for understanding and enacting quality in practice.

### 3.2 Orientation for action

The second category, orientation for action, concerns the approaches professionals use to achieve quality in practice and thus reflects how quality is operationalized through concrete actions. Similar to the previous category, two contrasting yet complementary orientations emerged: one emphasizing codified procedures and routines, and the other prioritizing adaptation.

#### 3.2.1 Codified procedures

The professionals described codified procedures as central to creating predictability and coherence across the organization. These included templates, checklists, and formalized workflows embedded in the quality management system. One document illustrates this orientation:

The management System includes identifying, describing, and establishing the processes within the organization that are necessary to ensure quality. A process refers to a series of activities that promote a specific purpose or intended outcome. (Service directive)

Such codifications were perceived as essential for aligning practice with shared standards and safeguarding uniformity across units and geographical boundaries. Formal documents described common frameworks and methods—such as standardized assessment tools—as means for promoting consistency in how services were intended to be provided to children and families.

#### 3.2.2 Adaptation

Simultaneously, professionals emphasized that quality emerges when attuning to the individual's circumstances. This perspective acknowledges that no universal solution can fully address the diversity of needs encountered in child welfare. Instead, quality was portrayed as co-created in the interaction between professional and client, requiring dialogue, relational competence, and flexibility.

Adaptation was described both as an overarching capacity to adjust to evolving situations and in terms of the specific, immediate adjustments made during encounters. Examples included tailoring interventions to the child's unique context. The professionals highlighted the importance of co-creation, describing how children and parents were actively involved in shaping the support they receive:

[…] the purpose is to develop and improve interventions together with the citizens we work with. They provide input on what we need to improve so that it benefits them, and we do this continuously so they can influence the process. (Clinical professional 3)

In their accounts, professionals described quality in ways that extend beyond procedural compliance, emphasizing engagement with children and families and responsiveness to their needs. Adaptation and co-creation were described as essential for interventions to be perceived as relevant. A similar emphasis was articulated in organizational documents, which highlighted building on the individual's own resources. As stated in the Quality management system:

The foundation of our management system is to build on the citizen's own abilities and resources to promote health. What we accomplish together should be experienced as meaningful, manageable, and comprehensible. (Quality management system)

#### 3.2.3 Navigating the dual orientation

The professionals did not perceive these orientations—the codified routines and adaptive practices—as mutually exclusive but as interdependent. Formalized workflows and standardized methods were described as necessary for ensuring predictability, legitimacy, and a shared direction across the organization. These structures provide what one professional called “a compass,” offering overarching guidance rather than rigid prescriptions. Yet, the professionals emphasized that strict adherence to detailed rules could be counterproductive, as every child and family presents unique circumstances that defy uniform solutions.

This balancing act required continuous judgment regarding when to follow established standards and when to diverge in favor of responsiveness. Several professionals articulated the tension between organizational expectations for consistency and the practical need for discretion. One practitioner explained:

Even if you create a template, many families don't fit into the template. But to be able to create more overarching frameworks for how to think about the work in different problem areas […] You can find support there and maybe it allows us to work in more similar ways. […] To me, frameworks are there to relate to a little more freely, and they give us a sense of direction, that this is how we want to work. (Clinical professional 3)

In these accounts, codified routines were described as providing guidance rather than detailed instructions. Professionals emphasized that frameworks were useful as points of reference, while still allowing flexibility in how work was carried out. Strategic professionals echoed this view, noting that overly detailed prescriptions risk undermining the ability to tailor interventions to individual needs:

Because we don't really know what it is, either, if we were to say, ‘How do you make a good assessment?’ And tell the employee: ‘This is what I expect,’ but how, you can't tell, it has to be adapted so much to the individual. It's not possible to have it, like, ‘Now we’ll always do it this way,’ then it ends up wrong, it becomes very wrong for some children and families. So we shouldn't get stuck in too detailed frameworks either, I think. (Strategic professional 6)

### 3.3 Value creation

Value creation describes what professionals see as the ultimate goal of their work—the type of value they aim to create through interventions in child welfare. Two distinct orientations emerged: one emphasizing political goals, and the other prioritizing life-changing impact for the individual.

#### 3.3.1 Quality as achieving political goals

In the organization's quality management system, achieving political and legal objectives was presented as a central aspect of quality. This perspective frames quality as compliance with official mandates, where fulfilling the assignments, requirements, and goals stipulated by laws and regulations is considered essential for safeguarding legitimacy and equality across units. The policy documentation states:

To develop and safeguard quality in the organization, we must fulfill assignments, requirements, and objectives as stipulated by laws and other regulations. (Quality management system)

This politically anchored view of quality was also voiced by one of the professionals in a strategic role, who emphasized adherence to legislation as the most fundamental definition of quality:

I basically follow what the National Board of Health and Welfare says in its regulations—that quality for us is to comply with the law. For me, that definition is the right one, no matter how much people try to complicate it. (Strategic professional 8)

In both interviews and documents, compliance with legislation was repeatedly identified as central to quality. References to economic considerations were less prominent in the interviews but appeared in organizational documents, such as requirements for reporting financial outcomes in the annual quality and results report.

#### 3.3.2 Quality as creating life-changing impact for the individual

In parallel, the organization's quality management system emphasized that quality improvement should lead to positive changes in individuals' lives. This ambition was echoed in the interviews, where professionals described quality as synonymous with achieving meaningful outcomes for children—such as ensuring safety, providing adequate support, and improving their living conditions. One clinical professional explained:

… and then also being able to help them, to actually make a change toward what the family, the parents, or the children want. That it really becomes a change. That's part of what I think of as quality in my work—being able to help them make the change they want. (Clinical professional 3)

Professionals also described the importance of listening to children and families, incorporating their views into planning and evaluation, and using tools such as feedback-informed approaches to ensure that interventions remain beneficial. As one strategic professional noted:

The basis for whether the work is of quality must be whether the people we are here to help feel helped. That has to be the ultimate measure of quality in some way. (Strategic professional 2)

However, the interviews also revealed the complexity of assessing such outcomes. Professionals described situations where their own assessment of positive change—such as improved school performance—did not necessarily align with the child's own priorities. A child might value autonomy over academic success, creating tensions between professional judgments and individual perspectives.

The professionals further reflected on the challenges of interpreting user feedback. A parent's satisfaction with the service, for example, might indicate good engagement, or it might signal insufficient attention to the child's perspective. Similarly, aggregated results from user surveys were described as providing only partial insight into children's and families' experiences.

#### 3.3.3 Navigating value tensions

The professionals described efforts to reconcile two competing value orientations: achieving political goals on the one hand, and creating meaningful, life-changing impact for individuals on the other. These orientations were not perceived as absolute opposites but as dimensions that coexist within the same practice, requiring continuous negotiation.

When quality was understood as uniformity, the creation of value centered on fulfilling societal mandates, such as compliance with legislation. During an analysis seminar, one participant remarked:

A sufficiently good quality is when we follow legislation and regulations—that's what the National Board of Health and Welfare says. (Analysis seminar participant A)

The participant viewed adherence to laws and formal guidelines as the primary indicator of quality. Other seminar participants challenged this view, pointing out that legal frameworks are open to interpretation and must be adapted to individual circumstances. This discussion illustrates that even within a compliance-oriented logic, flexibility is necessary to accommodate unique situations.

Conversely, when quality was framed as responsiveness, professionals emphasized the individual's well-being and long-term life changes. Clinical professionals described that organizational targets—such as reducing the duration of interventions or minimizing the number of placement changes for children—sometimes conflicted with what they considered best for the child. One practitioner explained:

We have a queue and hope to reduce the length of our cases so that we can take on more cases, and that is the kind of [goal] that is followed up. But then, on the one hand I can think that if I have a family with great difficulties and feel that this is not a quick fix, then I kind of ignore that goal because here I feel that no, this has to take its time. (Clinical professional 10).

Another professional reflected on the tension between minimizing placement breakdowns in foster care and acting in the child's best interest:

The goal is to reduce placement breakdowns, but not at any cost. Sometimes moving the child is the right thing to do. (Clinical professional 7)

In these examples the professionals described situations in which organizational priorities and performance targets did not align with the use of professional judgement when weighing organizational expectations against ethical and relational considerations in specific cases.

### 3.4 Quality as a negotiated and paradoxical construct in child welfare practice

**Figure 1:**
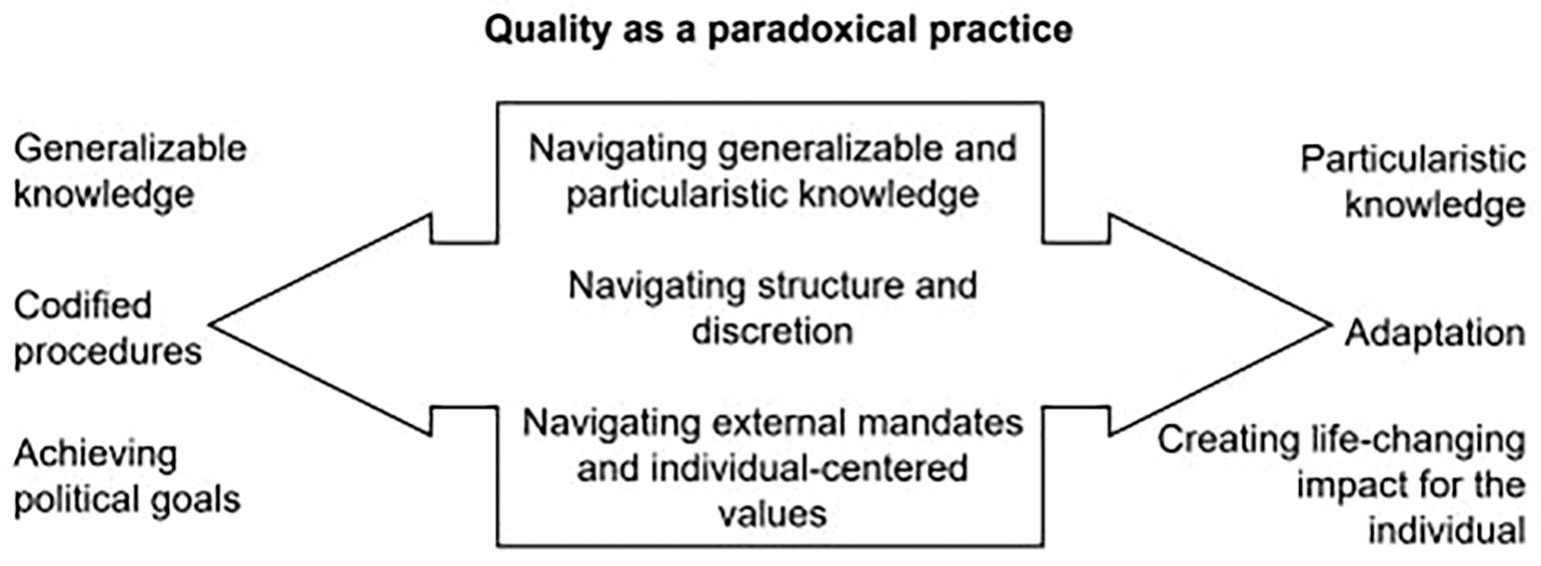
Synthesis of how the two logics of quality interact across three analytical categories, illustrating the paradoxical nature of practice where professionals draw on both logics simultaneously.

Ultimately, the professionals described quality as a negotiated construct rather than a fixed standard. Figure 1 provides an extended synthesis of the empirical findings, illustrating how the two logics of quality—uniformity and responsiveness—interact across the three analytical dimensions. The figure highlights the paradoxical nature of quality in child welfare practice, showing that professionals do not exclusively operate within one logic but continuously navigate and integrate both.

## 4 Discussion

This article has explored how child welfare professionals in Sweden understand and enact quality in their everyday work. In the following, the findings are discussed in relation to quality as a paradoxical practice in child welfare, and their implications for improvement science in complex human service systems.

### 4.1 Quality as a paradoxical practice in child welfare

The findings show that quality is not a fixed or singular concept but emerges through the interplay of two coexisting logics: one emphasizing uniformity, and the other privileging responsiveness to uniqueness. These logics do not simply compete; they coexist in a dynamic and persistent tension. They are not temporary operational dilemmas but enduring paradoxes that professionals navigate in their everyday work. Rather than eliminating one orientation in favor of the other, practitioners integrate both, using codified routines as a foundation while exercising discretion to adapt to unique circumstances. In this way, professional judgment becomes a critical resource for managing complexity.

The findings further demonstrate that tensions between uniformity and responsiveness oscillate between latent and salient states depending on contextual triggers. As Hahn and Knight (25) argue, paradoxes exist as latent potential within organizational systems and become salient when actors engage with them in specific situations. This perspective clarifies why tensions embedded in organizational policy documents surface as explicit challenges in practice. Our findings illustrate this dynamic: at the policy level, tensions remain largely latent, as policy documents articulate both logics of quality without explicitly addressing their contradiction. For example, the quality management system signals a codified, process-oriented approach aligned with uniformity, while simultaneously emphasizing adaptation and co-creation. Quality is thus framed both as procedural compliance and as relational responsiveness. Similarly, policy documents addressing value creation stress compliance with laws and regulations while also highlighting ambitions to achieve meaningful outcomes for children and families. Although both logics are formally acknowledged, their inherent tension is left unarticulated.

The latent paradox between uniformity and responsiveness becomes salient in practice when professionals are required to prioritize one logic over the other in concrete situations. The perceived coherence, suggesting that quality can be achieved through the simultaneous pursuit of both logics, tends to break down when organizational targets conflict with practitioners' assessment of what is best for the child. For example, targets aimed at reducing intervention duration may clash with practitioners' judgments that complex family situations require extended engagement. As shown in the findings, professionals sometimes deviate from organizational targets, such as reducing case duration or minimizing placement disruptions, in order to act in the child's best interest. In these situations, the tension between efficiency-oriented goals and responsiveness to individual needs become explicit and demands professional judgment. At the same time, standardized templates and workflows are described as essential for legitimacy and predictability, yet rigid adherence to such routines is perceived as undermining quality when unique circumstances require flexibility. From a paradox perspective, these situations illustrate how tensions embedded in organizational goals remain latent until they become salient in everyday practice, when professionals must actively navigate competing imperatives.

From a paradox perspective, these choices underscore why such tensions persist: they involve contradictory yet interdependent elements that cannot fully be resolved (21, 22). Hahn and Knight's (25) view further reinforces this point by showing how paradoxes materialize through interpretation and action, making them concrete challenges rather than abstract challenges. Importantly, these tensions extend beyond operational dilemmas to include structural features embedded within the organization's design and governance.

Taken together, the findings demonstrate that quality in child welfare is enacted through the ongoing coexistence of uniformity and responsiveness. While both logics are formally recognized in policy, their contradiction becomes salient in practice, requiring professionals to exercise judgment when organizational targets diverge from perceived needs for children and families.

### 4.2 Implications of paradoxical quality for improvement science

Understanding quality as a paradoxical practice highlights the limitations of some improvement approaches that rely on resolving competing demands by prioritizing one over another. Instead, the findings underscore the relevance of paradox thinking and to embracing interdependent contradictions. In this study, the tensions observed correspond to both performing and organizing paradoxes (21): conflicts between efficiency-oriented goals and individualized, long-term outcomes, and between codified routines and the adaptive work required in complex situations. These are not temporary dilemmas but can be understood as enduring conditions that shape how improvement unfolds.

Recognizing such tensions as persistent rather than solvable aligns with ideas associated with Quality 3.0 (19), particularly its emphasis on contextual sensitivity and co-production. This also resonates with research on complexity leadership and co-production which emphasizes that improvement in human service systems depends on the capacity to navigate, rather than eliminate, such competing demands (44). A paradox perspective extends this by showing that co-production and adaptive practices do not dissolve contradictions; instead they often bring them to surface as multiple perspectives and shifting conditions intensify paradox experiences (1). From the perspective of this study, strengthening quality may therefore involve combining reliable structures with adaptive capacities, and cultivating systems that support reflection on tensions, rather than treating variation or goal conflicts as implementation failures. In this sense, paradoxes can function as productive drivers of inquiry and learning in complex human service systems.

## 5 Conclusion and implications

### 5.1 Conclusion

This study demonstrates that quality in child welfare practice is shaped by a persistent paradox. The logics of quality as uniformity and quality as responsiveness to uniqueness do not represent competing alternatives but coexist in dynamic tension, continuously negotiated by professionals. Rather than being anomalies, such tensions are constitutive of practice, consistent with paradox theory's view that paradoxes involve elements that are both contradictory and interdependent, coexisting over time despite their opposition (21). Quality thus emerges as a relational and interpretive phenomenon rather than a fixed attribute. This understanding highlights the complexity of child welfare, where professionals continuously balance policy demands with their assessment of children's and families' needs.

By foregrounding quality as a paradoxical practice, the study contributes to a deeper empirical understanding of how quality is enacted in child welfare. While grounded in this specific context, the findings point to broader challenges faced in complex human service systems, where improvement efforts often need to work with enduring tensions rather than resolve them definitely.

### 5.2 Implications

While this study primarily contributes to knowledge about quality in child welfare, the findings also have implications for improvement science as applied in complex human service systems. Rather than treating tensions as problems to be eliminated, improvement approaches may benefit from explicitly acknowledging paradox as a persistent condition of practice. Specifically, the findings suggest that improvement strategies could be informed by:

- Structures, such as reflective forums, that make tensions discussable and facilitate competing understandings of quality to be articulated rather than resolved.
- Organizational designs that legitimize professional discretion within quality improvement work, for example by recognizing judgment-based deviations as integral to improvement processes rather than exceptions to be corrected.
- Follow-up practices that tolerate ambiguity, allowing space for professional judgment alongside technical and measurable aspects of improvement.
- Co-production arrangements that acknowledge that collaboration across roles and perspectives might heighten tensions around quality and that such tension still can be productive drivers for improvement.

These implications do not prescribe specific solutions. Instead, they highlight how a paradox perspective can inform the design and use of quality improvement approaches that are sensitive to the relational and contextual realities of complex human service systems. Future studies could explore similar tension dynamics in other quality improvement initiatives where such tensions remain underexamined.

## Data Availability

The raw data supporting the conclusions of this article will be made available by the authors, without undue reservation.
